# Factors associated with the inclusion of oral health technicians into the public health service in Brazil

**DOI:** 10.1186/s12960-019-0371-7

**Published:** 2019-05-24

**Authors:** Ana Cláudia Pereira dos Santos Cruz, Simone Dutra Lucas, Lívia Guimarães Zina, Rafaela da Silveira Pinto, Maria Inês Barreiros Senna

**Affiliations:** Universidade Federal de Minas Gerais, Belo Horizonte, MG Brazil

**Keywords:** Dental hygienists, Allied health personnel, Health workforce, Health manpower, Health management, Public oral health

## Abstract

**Background:**

The number of oral health technicians (OHT) in the public health service in Brazil is lower than the number of training school graduates. Thus, the objective of this study was to investigate possible factors associated with the inclusion of OHT in the public health service in Minas Gerais, Brazil, and its implications on oral health indicators.

**Methods:**

This cross-sectional ecological study used a database (Excel) composed of 122 municipalities that participated in an OHT training course that took place between 2012 and 2013. Municipal contextual variables, including oral health indicators and sociodemographic indicators, related to the organization of health services were incorporated before and after the course. The dependent variable was the entry of graduates into the public health service according to a self-report survey conducted in 2015. A descriptive analysis of the variables was carried out, followed by bivariate analyses between the independent variables and the dependent variable using Pearson’s chi-square test. The independent variables selected for multivariate logistic regression were statistically significant at *p* <  0.20. In the final model, significant effects were identified for variables with *p* <  0.05. The statistical software SPSS 18.0 for Windows was used.

**Results:**

After the course, the variable of the public service organization and the two variables of oral health indicators were associated with the outcome. The organization services variable “presence of oral health team modality II” and the variable “indicator of coverage of first dental programmatic consultation” presented an association tendency with the entry of OHT in the multivariate logistic regression model, but these associations were not statistically significant because they had significance levels of *p* = 0.075 and *p* = 0.191, respectively. The variable “collective action indicator supervised dental brushing” was associated with the entry of egress (*p* = 0.045) remaining in the final model.

**Conclusion:**

The model of organization of the oral health service formed through the implementation of modality II oral health teams positively influenced the inclusion of OHT in the public health service in Minas Gerais, with improvement in the oral health indicators of the municipalities.

## Introduction

The importance of human resources for the formulation of public policies on health is unquestionable since professionals qualified to work in this sector play a fundamental role in the realization or maintenance of most functions of health care systems. The main aspects that should be analysed by health service managers are the formation of a qualified workforce and its distribution, which concerns the recruitment and retention of the workers where they are most needed and their performance, i.e. the quality of care provided [1]. In terms of training, substantial time and resources have been invested worldwide for qualified professionals in the health sector [2].

An oral health technician (OHT) is a health worker in Brazil with a profile similar to that of dental hygienists and dental therapists in various regions of the world. For example, dental hygienists are present in the United States (US), Israel and the United Kingdom and are workers who carry out preventive actions in oral health. Dental therapists have expanded functions in relation to hygienists, such as performing restorative procedures, and they work in several countries, such as Australia, Canada, Alaska and in some US states [3–11]. In Brazil, the regulation of professional practice occurred in 2008, establishing that OHT can act only under the supervision of the dental surgeon [12]. Globally, there is a great difference between countries in relation to the regulation and autonomy of these human resources; in the US, they have considerable independence or a more restricted performance in accordance with state legislation [3, 9, 13, 14]. In 2017, Brazil had 13 OHT per 100 000 inhabitants enrolled in the federal body responsible for the supervision and regulation of dentistry [15]. The OHT comprise the dental health team (DHT) of the family health team (FHT) of the Brazilian Public Health System (SUS). The National Policy of Basic Attention [16] establishes the following attributions for OHT: to train oral health auxiliaries (OHAs) and health promoters; to welcome patients in oral health services; to carry out programme activities with attention to spontaneous demand; to participate in surveys and epidemiological studies; to monitor, support and develop collective activities related to oral health with other members of the team, seeking to approach and integrate health actions in a multidisciplinary way; and to perform clinical care, acting to promote health and prevent oral diseases. OHT are responsible for actions regarding prevention, protection, promotion and the recovery of individuals’ health at the individual and collective levels [16]. OHT are fundamental actors in expanding access to health care and reducing health disparities, and OHT inclusion in the DHT is aimed at rationalizing work and increasing productivity and quality, aiming to change the practices and models of oral health care [13, 16–19]. The composition of the DHT in the FHT was defined by Ordinance No. 1444 of the year 2000 by the Ministry of Health (MH) [20] and established two modalities of the DHT: modality I is composed of a dentist and an OHA, and modality II is formed by a dentist, an OHA and an OHT. For the entry and formal performance of the OHT, it is necessary for municipal health services to establish modality II DHT.

The technical training of workers for public health services in Brazil is carried out by 40 schools that make up the Network of Technical Schools of the Public Health System (RET-SUS). The RET-SUS is a governmental network that aims to meet the demands of human resources in health, such as the OHT, using the training of human resources as a strategy through teaching-service integration [21].

The study of the training and entry of dental workers in the labour market, especially in the public sector and in countries with models of social insurance or socialization of health services, contributes to the planning and execution of preventive and assistance actions aimed at access to public oral health services [17].

Zina et al. [22] analysed the entry patterns of OHT graduates from the School of Public Health of the State of Minas Gerais (ESP/MG) of the RET-SUS network, and the results showed that only 10% of respondents were employed as OHT in the SUS. This finding indicated a need to investigate the factors associated with OHT entry in the state of Minas Gerais. This state, located in the southeastern region of the country, is one of the 27 federated units of Brazil, with the fourth largest territorial area (586 528 km^2^), the largest number of municipalities (853) and the second largest population (21.1 million inhabitants). In 2017, it contained 80.91% of the FHT population and 34.5% of the DHT population. Of the DHT, 50.6% are classified as modality I and 49.4% are classified as modality II, representing the highest population of modality II DHT in the country (1450). The region’s rate of OHT enrolled in the Federal Council of Dentistry (CFO) is above the national average (19 per 100 000 inhabitants) [15, 23].

Studies addressing the entry of OHT into the public health service and contextual characteristics of municipalities and health services were not found in the reviewed literature. The focus of research involving these human resources has been the characterization of the graduates (sociodemographic profiles) and their entry into the labour market [24–28].

The objective of this study was to investigate the possible factors associated with the entry of OHT in the public health service in Minas Gerais, Brazil, and its implications on oral health indicators.

## Methods

A cross-sectional ecological study was carried out in the state of Minas Gerais, Brazil, using a database from the research project “Evaluation of the Impact of Training of Oral Health Technicians in the Public Health Network in the State of Minas Gerais”, with 618 graduates of OHT courses offered between 2012 and 2013 by ESP/MG [22]. As an ecological study, the universe of study included 248 municipalities that sent workers to qualify to be OHT between 2012 and 2013. The sample size was calculated in 151 municipalities, adopting a confidence level of 95%. The selection of the participating cities was determined via the graduates who were interviewed in the study by Zina et al. [22], totalling 122 municipalities. There was a sample loss of 19%.

The dependent variable of the study was the “insertion of OHT into the public health service” (yes/no) according to respondents’ answers to the interview conducted in 2015 [22]. The graduates were identified according to the code used by the Brazilian Institute of Geography and Statistics (IBGE) for the municipalities in which they worked at the time they attended the course. The independent variables addressed three dimensions of the municipal context, the organization of health services, indicators of oral health in primary health care and sociodemographic conditions as presented in Table 1.

- Health service organization variables: The choice of variables “population coverage of Family Health Strategy”, “population coverage of Oral Health Teams”, “proportion between Oral Health Teams and Family Health Teams” and “presence of Oral Health Team modality II” was based on government ordinances that defined the parameters for the organization of health services within the scope of primary health care (PHC) [20, 29, 30, 31]. The variable “proportion of dentists per 1,000 inhabitants” was chosen with the purpose of defining the concentration of these professionals considering that performance of OHT is linked to the supervision of a dental surgeon [12].
- Indicators of oral health: Two indicators established by the Basic Attention Pact, a reference instrument for monitoring and evaluating actions developed within the country’s PHC system [32], were selected: (1) “coverage of first programmatic dental consultation”, which corresponded to the percentage of people who received an initial consultation conducted for the purpose of diagnosis and necessity, elaboration of a preventive therapeutic plan, and (2) “coverage of group activities such as supervised dental brushing”, corresponding to the average number of people who had access to dental brushing with orientation or supervision by a trained professional considering the month or months in which the activity was carried out, in a specific place and year, aiming at the prevention of oral diseases, specifically dental caries and periodontal disease [32]. These indicators were used in the state of Minas Gerais as criteria for approving specific financing for DHT [35, 36].
- Sociodemographic variables: The “Municipal Human Development Index (MHDI)” was selected, which is a measure made up of indicators of three dimensions of human development: longevity, education and income [37], and the “Gini index” used to measure the concentration of income in a given group [38]. Previous studies have found a correlation between indicators of socioeconomic development and indicators of oral health and the allocation of human health resources [2, 27, 39, 40]. The variable “population size” was chosen based on literature findings that suggested that the number of inhabitants of a municipality can influence the health care model offered to the population [41, 42]. Data were collected from public domain databases and Brazilian governmental electronic sites (Department of Basic Attention (DAB), Department of Information Technology of the Public Health System (DATASUS) and IBGE) [23, 33, 34]. The dimensions of the Health Service Organization and Oral Health Indicators refer to the years 2011 (before the OHT training course) and 2014 (after the inclusion of the OHT). The municipal sociodemographic data refer to the year 2010. The variables were dichotomized according to the reference parameters recommended by the MH. The median was calculated by the authors for Minas Gerais; the values for the state were already calculated.

**Table 1:** Dependent and independent variables of the study

Contextual dimensions		Variables	Categories	Reference year	Data sources	Rationale for categorization
Dependent variable		Entry of OHT into the public service	Yes No	2015	Search database	Based on the results of the research “Evaluation of the impact of training of Oral Health Technicians in the public health network in the state of Minas Gerais” [22].
Independent variables	Health services organization	Population coverage of FHT	< 100%	2011	DAB	Ideally the coverage should be 100% [29].
			= 100%	2014		
		Population coverage of DHT	< 70%	2011	DAB	Median of the state of Minas Gerais
			≥ 70%	2014		
		DHT/FHT ratio	< 70%	2011	DAB	Median of the state of Minas Gerais
			≥ 70%	2014		
		Presence of DHT modality II	Yes	2011	DATASUS	Has or does not have OHT
			No	2014		
		Proportion of dentists per 1 000 inhabitants	< 0.67	2011	DATASUS	Calculation for 1 000 inhabitants, based on the recommendation of the old Ordinance n° 1 101 of the dentist for at least 1 500 inhabitants [30].
			≥ 0.67	2014		
	Oral health indicators	Indicator of coverage of the first dental programmatic consultation	< 15%	2011	DATASUS	National reference value of the Basic Attention Pact [32].
			≥ 15%	2014		
		Indicator of coverage of the collective action of supervised dental brushing	< 3.5%	2011	DATASUS	National reference value of the Basic Attention Pact [32].
			≥ 3.5%	2014		
	Sociodemographic variables	MHDI	< 0.731	2010	IBGE	HDI value of the state of Minas Gerais [33].
			≥ 0.731			
		Gini index	< 0.5634	2010	DATASUS	Median of the state of Minas Gerais
			≥ 0.5634			
		Population size	< 10.000	2010	IBGE	44.54% of the municipalities of Minas Gerais have less than 10 000 inhabitants [34].
			≥ 10.000			

The data were exported from the Excel 2010 programme to the statistical programme Statistical Package for the Social Sciences (SPSS) 18.0 for Windows. A descriptive analysis was performed with frequency calculations. Bivariate analyses were performed between the outcome and the health service organization variables, oral health indicators for the years 2011 (before the course) and 2014 (after the course) and sociodemographic variables for the year 2010 using Pearson’s chi-square test. Then, we selected the independent variables that were statistically significant (*p* <  0.20) for the bivariate and multivariate logistic regression analyses between the outcome and the independent variables. In the final model, those presenting a value of *p* <  0.05 were considered statistically significant.

## Results

**Fig. 1:**
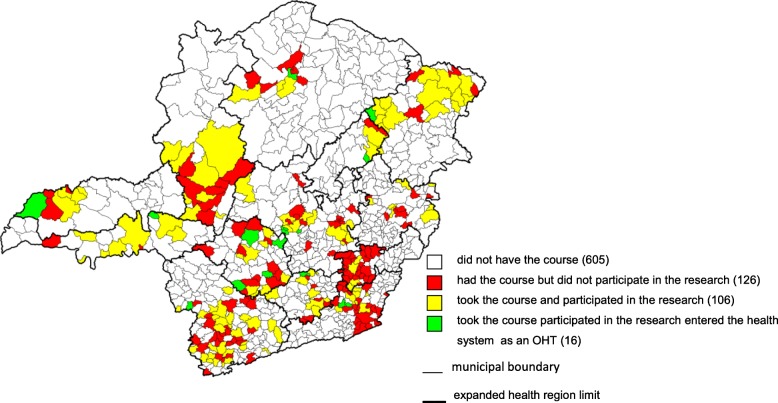
Situation of municipalities in relation to their participation in the oral health technician course, in the research and entry into the public health service, Minas Gerais, 2015

Figure 1 shows the spatial distribution of the municipalities of Minas Gerais and their relation with the egress of the OHT course. Noticeably, the educational programme offered between the years 2012 and 2013 presented a wide geographic distribution in Minas Gerais since it included all of the expanded regions of health (ERH) in the state. The ERHs are territories delimited for the planning of health care, which consider demographics, socioeconomics, geography, health, epidemiological characteristics, service provisions and relations between municipalities, among others [43]. During that period, 248 cities representing 29% of the municipalities of Minas Gerais sent students to the course. This percentage can be considered high, and since the course is offered periodically, one can predict that the future trend is for all municipalities to benefit. The 122 municipalities that constituted the sample of this study represented 49.2% of the course participants. However, the percentage of OHT that entered the public health service represents only 15% of the municipalities. These municipalities are located in the central and southern regions of the state of Minas Gerais and are more economically developed regions.

Between 2011 and 2014, the percentage of municipalities with FHT coverage equal to 100% increased by 15.6%. A slight increase of 9% was also observed for municipalities with DHT coverage greater than or equal to 70%. As a consequence, the frequency of municipalities with a ratio of DHT to FHT greater than or equal to 70% also increased (4.9%), along with the frequency of municipalities with modality II DHT, which increased by 4.1%. The frequency of municipalities with a proportion of dentists per 1 500 inhabitants greater than or equal to 0.67 decreased by 1.6%. Regarding oral health indicators, there was a decrease in the number of municipalities that reached the recommended goal: 11.5% for coverage of the first dental visit and 1.6% for supervised dental brushing coverage in the same period. Most of the municipalities studied had a lower MHDI and Gini index than those of Minas Gerais (0.731), i.e. the majority of municipalities studied had a poorer quality of life and greater inequality in income distribution. It should be noted that the majority (67.2%) of the municipalities participating in the survey were municipalities with a population greater than 10 000 inhabitants (Table 2).

**Table 2:** Frequency distribution of the context variables of the municipalities surveyed before and after the OHT course, Minas Gerais, Brazil, 2014

	Municipal variables	2011		2014	
		*n*	%	*n*	%
Health services organization	Population coverage of FHT				
	< 100%	89	73.0	70	57.4
	= 100%	33	27.0	52	42.6
	Population coverage of DHT				
	< 70%	73	59.8	62	50.8
	≥ 70%	49	40.2	60	49.2
	ESB/ESF ratio				
	< 70%	62	50.8	56	45.9
	≥ 70%	60	49.2	66	54.1
	Presence of DHT modality II				
	Yes	33	27.0	38	31.1
	No	89	73.0	84	68.9
	Dentists per 1 000 inhabitants				
	< 0.67	83	68.0	81	66.4
	≥ 0.67	39	32.0	41	33.6
Oral health indicators	First consultation				
	< 15%	77	63.1	91	74.6
	≥ 15%	45	36.9	31	25.4
	Supervised dental brushing				
	< 3.5	66	54.1	68	55.7
	≥ 3.5	56	45.9	54	44.3
Sociodemographic indicators		2010			
		*n*		%	
	MHDI				
	< 0.731	102		83.6	
	≥ 0.731	20		16.4	
	Gini Index				
	< 0.5634	114		93.4	
	≥ 0.5634	8		6.6	
	Population size				
	< 10 000 inhabitants	40		32.8	
	≥ 10 000 inhabitants	82		67.2	

Data sources: DAB, DATASUS, IBGE

The results showed that sociodemographic variables did not present statistical significance (*p* > 0.05), suggesting that in this study, the MHDI, Gini index and population size were not factors associated with the inclusion of OHT in the public oral health service for the municipalities surveyed (Table 3).

**Table 3:** Frequency distribution of sociodemographic variables according to the entry of OHT into the public health service in the participating municipalities, Minas Gerais, Brazil, 2010

Variables	Categories	2010				
		Entered		Not entered		p*
		*n*	%	*n*	%	
MHDI						
	< 0.731	13	12.7	89	87.3	0.785
	≥ 0.731	3	15.0	17	85.0	
Gini index						
	< 0.5634	15	13.2	99	86.8	0.985
	≥ 0.5634	1	12.5	7	87.5	
Population size						
	< 10 000 inhabitants	7	17.5	33	82.5	0.316
	≥ 10 000 inhabitants	9	11.0	73	89.0	

*Chi-square test

Data sources: DATASUS, IBGE

For the year 2011, no significant associations were found, with *p* <  0.20. The same analyses were carried out for the year 2014, and three variables that showed a statistically significant association (*p* <  0.20) were included in the logistic regression model: “presence of DHT modality II”, “indicator of coverage of first programmatic dental consultation” and “indicator of supervised dental brushing” (Table 4).

**Table 4:** Bivariate analysis between the health service organization variables and oral health indicators according to the entry of the OHT into the public health service in the participating municipalities, Minas Gerais, Brazil, 2014

	Variables	2011					2014				
		Entered		Not entered		p*	Entered		Not entered		p*
		*n*	%	*n*	%		*n*	%	*n*	%	
Health services organization	Population coverage of FHT										
	< 100%	11	12.4	78	87.6	0.685	10	14.3	60	85.7	0.657
	= 100%	5	15.2	28	84.8		6	11.5	46	88.5	
	Population coverage of DHT										
	< 70%	9	12.3	64	87.7	0.754	7	11.3	55	88.7	0.544
	≥ 70%	7	14.3	42	85.7		9	15.0	51	85.0	
	DHT/FHT ratio										
	< 70%	9	14.5	53	85.5	0.641	5	8.9	51	91.1	0.207
	≥ 70%	7	11.7	53	88.3		11	16.7	55	83.3	
	Presence of DHT modality II										
	No	11	12.4	78	87.6	0.685	7	8.3	77	91.7	0.020
	Yes	5	15.2	28	84.8		9	23.7	29	76.3	
	Dentists per 1 000 inhabitants										
	< 0.67	12	14.5	71	85.5	0.521	10	12.3	71	87.7	0.724
	≥ 0.67	4	10.3	35	89.7		6	14.6	35	85.4	
Oral health indicators	First consultation										
	< 15%	9	11.7	68	88.3	0.541	9	9.9	82	90.1%	0.071
	≥ 15%	7	15.6	38	84.4		7	22.6	24	77.4%	
	Supervised dental brushing										
	< 3.5	8	12.1	58	87.9	0.724	4	5.9	64	94.1%	0.008
	≥ 3.5	8	14.3	48	85.7		12	22.2	42	77.8%	

* Chi-square test

Data sources: DATASUS, IBGE

A multivariate logistic regression analysis was performed for 2014 (Table 5) between the outcome and the independent variables that remained significant at *p* <  0.20. In the final model, associations with values of *p* <  0.05 were considered statistically significant, with “supervised dental brushing indicator” remaining. The variables “indicator of coverage of first programmatic dental consultation” and “presence of DHT modality II” showed association tendencies that were not statistically significant.

**Table 5:** Unadjusted and adjusted analyses for the variables of organization of health services and indicators of oral health for the year 2014, Minas Gerais, Brazil, 2014

Variable	Category	2014			
		Unadjusted OR (95% CI)	*p*	Adjusted OR (95% CI)	*p*
Organization of health services	Presence of DHT modality II				
	No	Ref	0.025	Ref	0.075
	Yes	3.41 (1.16–10.01)		2.76 (0.90–8.50)	
Oral health indicators	Frist consultation				
	< 15%	Ref	0.078	Ref	0.191
	≥ 15%	2.65 (0.89–7.88)		2.15 (0.68–6.80)	
	Supervised dental brushing				
	< 3.5	Ref	0.013	Ref	0.045
	≥ 3.5	4.57 (1.38–15.12)		3.53 (1.02–12.14)	

*OR* odds ratio, *CI* confidence interval, *Ref* reference category

Data sources: DAB, DATASUS

## Discussion

The sociodemographic variables did not present a statistically significant association with OHT inclusion into the public health service and with the oral health indicators. This can be explained by the fact that in the sample studied, the number of OHT included into the public health service was small. This result differs from the study of Fernandes and Peres [40], conducted in 293 municipalities in the state of Santa Catarina, Brazil. This research evaluated the correlation between oral health indicators and socioeconomic indicators of the municipalities and concluded that the better the municipal socioeconomic indicators were, the better the oral health indicators were. In a more global context, Kabene et al. [2] analysed the correlation between the distribution of health professionals and sociodemographic indicators in the health systems of Canada, the US, Germany and several other developing countries using secondary data and qualitative analysis. The results showed that the better these indicators were, the greater the incorporation of professionals into the public health service [2].

In the bivariate logistic regression model, it was verified that a municipality with modality II DHT had a 3.41 times increased chance of OHT inclusion. Although this variable did not remain significant in the multiple regression model, the formal inclusion of OHT into DHT is related to the habilitation of modality II DHT. The establishment of modality II DHT by a municipality is an important step to improve the oral health care model since it allows OHT to work in basic health units. Care and bonding constitute the center line of their actions, favouring the expansion of health practice aimed at the humanization of care [44]. It is important to emphasize that the federal government has financial incentives for the implementation of DHT and that the financial resources for modality II DHT are greater than those for modality I [45]. Mattos et al. [18] analysed the inclusion of DHT in FHT in Minas Gerais and found that federal resources were the main motivators for municipal management to enable the implementation of modality II DHT. The establishment of modality II DHT is related to the greater knowledge of municipal managers regarding the importance of OHT in the improvement of the effectiveness of the DHT in public oral health [19].

Bonan et al. [25] found that only 7.8% of graduates from another RET-SUS school in Minas Gerais worked as OHT in the FHT, and this result is similar to that of the study by Zina et al. [22]. Lima et al. [19] observed that 20.7% of the graduates from a RET-SUS school in Ceará worked as OHT in the public health service. It should be noted that the state of Ceará has a proportion of 8% of modality II DHT in relation to the number of DHT implanted, unlike in Minas Gerais, in which the proportion reaches 23% [23]. These national studies indicate that the number of RET-SUS school OHT graduates is higher than the number of OHT enrolled in the public oral health service. In the Brazilian context, technical training has received a large volume of financial investment from the SUS. The training of 618 OHT carried out by ESP/MG in 2012 and 2013 had a cost of approximately 450 000 euros [46].

The National Oral Health Policy [44] proposes joint work between the DHT and the FHT. In this context, the inclusion of OHT in the DHT within the FHT represents a key element that contributes to assistance actions and the expansion of the access to health services [13, 17, 18]. Since the implementation of this policy, there has been a significant intensification of government funding for the training of oral health auxiliary staff for the public sector, mainly since the inclusion of DHT in the FHT [13, 17, 25]. The non-incorporation of OHT makes it difficult to expand the coverage as the reconfiguration in the human resources framework of health services. The inclusion of OHT in the public health service has promoted the expansion of access to oral health care, especially for children in New Zealand, Australia, the United Kingdom, Canada and the US [47, 48], which confirms the premise that this worker contributes to the increase in coverage and the decrease in the iniquities in oral health.

The incorporation of OHT has the potential to favour an increase in the first dental programmatic consultation and impacting this indicator since OHT can perform preventive clinical procedures such as dental plaque removal, calculation scaling and fluoride application. The results were not significant for this variable in the final model, which seems to indicate an underutilization of the OHT’s clinical competence. These findings concur with the study by Sanglard-Oliveira et al. [49] on the role of OHT in the SUS in Minas Gerais. The authors found a lower possibility of the OHT contribution in individual clinical care, mainly in restorative care in FHT in Minas Gerais, but with more frequent action in collective activities.

The collective procedures more frequently performed by OHT in the public health service in Brazil are supervised dental brushing and topical fluoride application [13]. Municipality with OHT inclusion in DHT had a 3.5 times greater chance of reaching the reference goal for this indicator when compared to those which did not include OHT.

The role of OHT in the FHT has a focus on the prevention of oral diseases and the promotion of health knowledge and practices. In addition to clinical performance, OHT have the function of participating in the process of planning, monitoring and evaluating actions carried out in the scope of the territory. It is within their competence to identify the needs and expectations of the population regarding oral health and encourage and implement health promotion measures, educational programs and preventive activities. OHT should organize the work process according to the guidelines of the SUS, educate families to the importance of oral health in the maintenance of health, schedule and carry out home visits according to identified needs and develop intersectoral actions for the promotion of health [49]. In the US, the practice of dental hygienists is directed towards individual preventive clinical care, which has generated studies that recommend a reform in the training and attributions of this human resource [9, 14].

The evaluation of a human resources policy within the public health service should articulate two systems: the production of human resource, which is the training for work, and the system of utilization of human resources, the labour management [50]. The management of human resource plays a significant role in the distribution of health professionals [2].

Global initiatives related to the assessments of the impact of government investments allocated to the training and utilization of human resources for health have been implemented, such as the Global Strategy for Human Resources for Health - Workforce 2030 and the Project aimed to policy makers and planners in the Member States of the World Health Organization. In adopting the Resolution No. 69.38 of 2006, all 194 member states, including Brazil, were requested to develop bilateral and multilateral initiatives to carry out investment-related assessments for human resources for health [51]. Although there are no standard tools for this type of evaluation, there are other types of strategies, such as research on this subject, which can and should be used by public and private sector employers, professional associations, training institutions, trade unions, international organizations and civil society [2, 52]. There is a small number of publications involving the training and work of oral health human resources in the public service and their contributions to reorganize the care model and improve the oral health conditions of the Brazilian population.

The use of a secondary source to collect data of the OHT inclusion was a limitation of this study. The data were obtained through self-reports from the graduates and for this reason may be underestimated due to the sample loss, not allowing a generalization of the results; however, the sample is representative for the state of Minas Gerais. The values obtained in the statistics of the final model were very close to the reference value of 0.05, and the importance of the findings for the organization of health services is considered since they indicate an improvement in the indicators. According to the literature and empirical observations, it is considered that the significance of OHT incorporation in public health services cannot be analysed solely for statistical significance, especially regarding the importance of their inclusion in the health care service for users and for policy makers [53, 54].

So that the inclusion of OHT in the public oral health service to be consistent with the volume of government financial investments spent on OHT training, changes related to training use must be made. It is necessary that the changes are initiated by political management in the municipalities. Such changes should be based both on the organization of the oral health service, particularly on the implementation of modality II DHT, as well as on the contracting and bonding of workers with the health system. Regarding the implantation of modality II DHT, it is observed that there is a trend of expansion of this type of modality today in Brazil [21]. This is justified by the ratio of DHT/FHT, which in most municipalities is one DHT to two FHT. Thus, in modality I DHT, a single dental surgeon is responsible for a large number of people. Lourenço et al. [55] carried out an investigation with these professionals who reported difficulty in carrying out promotion and prevention activities due to the great demand for curative actions, explained by the health care debt to which the population was submitted to throughout history and by the lack of programmes. In the modality II DHT, the OHT share responsibilities with the dental surgeon, which can be realized in the improvement of the oral health indicators [55]. With regard to the management of human resources in health, several studies point to a shortage of public tenders to fill vacancies for this position, creating precarious links and making oral health actions more difficult since employees are not guaranteed long-term permanence [18, 21, 55–57]. The perception of all the actors involved in the formation and inclusion of OHT is very important for understanding the factors that interfere with the incorporation of this human resource in public health services. We conducted a survey with the teachers of the course (data not yet published), and we realized the need to continue the study seeking to understand the perception of municipal managers through a qualitative approach.

## Conclusion

The model of organization of the oral health service formed through the implementation of modality II oral health teams has positively influenced the inclusion of OHT in the public health service in Minas Gerais, with an improvement in oral health indicators of the municipalities.

## Abbreviations

CFO: Federal Council of Dentistry
DAB: Department of Basic Attention
DATASUS: Department of Information Technology of the Public Health System
DHT: Dental health team
ERH: Expanded regions of health
ESP/MG: School of Public Health of the State of Minas Gerais
FHT: Family health team
IBGE: Brazilian Institute of Geography and Statistics
MH: Ministry of Health
MHDI: Municipal Human Development Index
OHA: Oral health auxiliaries
OHT: Oral health technician
PHC: Primary health care
RET-SUS: Network of Technical Schools of the Public Health System
SPSS: Statistical Package for the Social Sciences
SUS: Public Health System
US: United States
